# Calcium ions decrease water-soaking in strawberries

**DOI:** 10.1371/journal.pone.0273180

**Published:** 2022-08-15

**Authors:** Grecia Hurtado, Moritz Knoche

**Affiliations:** Institute for Horticultural Production Systems, Leibniz-Universität Hannover, Hannover, Germany; Universidade do Minho, PORTUGAL

## Abstract

Water soaking is a common disorder of field-grown strawberries (*Fragaria × ananassa* Duch.). It develops when ripe fruit is exposed to rain. Here we investigate the effects of Ca on water soaking. Fruit was incubated in solutions of various Ca salts and the extent of water soaking quantified using a simple rating scheme. Exposure to CaCl_2_ (10 mM) decreased water soaking and anthocyanin leakage but had no effect on water uptake. The decrease in water soaking due to CaCl_2_ was not limited to a single cultivar but occurred in all cultivars examined. Incubating fruit in a chelating agent (EGTA) increased water soaking compared to the water control. Calcium salts of different acids varied in their effects on water soaking. Only CaCl_2_ reduced water soaking significantly. The chlorides of different cations, also varied in their effects on water soaking. Those of the monovalent cations had no effects on water soaking, while those of the divalent cations (CaCl_2_, BaCl_2_ and SrCl_2_) and of the trivalent cations (FeCl_3_ and AlCl_3_) were all effective in decreasing water soaking. Overall, CaCl_2_ decreased microcracking of the strawberry cuticle as compared to deionized water. Furthermore, CaCl_2_ also reduced the leakage of anthocyanins from flesh discs, irrespective of the osmotic potential of the incubation solution. Our results indicate that CaCl_2_ reduced water soaking by decreasing cuticular microcracking, by decreasing leakage of plasma membranes and, possibly, by increasing the crosslinking of cell wall constituents.

## Introduction

Water soaking is an economically important disorder of field grown strawberries [1]. It develops when fruit is exposed to rain during ripening. Protected cultivation in greenhouses or tunnels covered with plastic film generally avoids water soaking [2] but it markedly increases the cost of production. Other countermeasures for water soaking are not known.

Recently, the physiological background of water soaking has been identified [2]. In many ways the process of water soaking in strawberry resembles the process of rain cracking in sweet cherry. Both, water soaking and rain cracking are multistep processes that can be described using the analogy of a ‘zipper’, that unzips the fruit skin [3]. Based on the ‘Zipper’ model [3], water soaking is initiated by the formation of microscopic cracks (‘microcracks’) in the cuticle [2]. Microcracking impairs the barrier function of the cuticle thereby permitting localized water uptake. When water uptake exceeds a critical limit, cells begin to burst [4]. Cell contents, including anthocyanins and organic acids, are thus released from the vacuole and move into the cell wall space. Strawberry is particularly rich in malic and citric acids [5]. Exposure to these acids increases the permeability of the membranes of adjacent cells. This causes water soaking to spread tangentially over the skin and also radially down into the flesh [2].

The above sequence of events is largely identical to the early steps of rain cracking in cherry. Also, the countermeasures taken against rain cracking in sweet cherry are similar to those taken in strawberry, i.e., production in protected environments using rain shelters or tunnels [3]. In addition, in sweet cherry, whole canopy sprays of Ca salts are reported to decrease susceptibility to cracking [6]. For strawberry, there is no published information on the possible benefits of whole canopy Ca sprays on water soaking of the fruit. However, given the hypothetical mode of action of Ca in decreasing rain cracking susceptibility in cherry, such beneficial effects are not unlikely with water soaking in strawberry.

The objective of our study was to establish the effects of Ca on water soaking in strawberry. The effects of Ca were compared to those of other monovalent, divalent and trivalent cations. Water soaking was induced using a laboratory based immersion assay [2].

## Materials and methods

### Plant material

Strawberry fruit were harvested from commercial plantings at Gleidingen (lat. 52°16´ N, long. 9°50´E), Ohndorf (lat. 52°21´ N, long. 9°21´E) and from a greenhouse and growth chamber on the Herrenhausen Campus of the Leibniz University, Hannover, Germany. Temperature and relative humidity (RH) of the growth chamber were set at 20/16°C and 60/80% RH during a 16 h day/night photoperiod. The cultivars used in our study were ‘Clery’, ‘Sonsation’, ‘Malwina’, and ‘Faith’. These cultivars were selected based on uniformity of fruit and availability at the optimum stage of ripening. The change of cultivars ensures that the observed effects are valid for strawberry in general and not limited to a specific cultivar. Fruit were harvested randomly at commercial ripeness (>80% of the fruit surface red) and selected for uniformity of size, shape, color, and freedom from visual defects. Earlier studies established that at this stage of development ripeness has no effect on the rate of water uptake [4] or on water soaking [2]. Fruit were processed fresh on the day of harvesting or held at 2°C and 80% RH for no longer than 1 d.

### General procedure

Unless otherwise specified, the so-called ‘calyx’ was removed from the fruit (strictly a false fruit) by carefully tearing away individual bracts. Fruit mass was sufficiently high that there was no need to hold down (and potentially damage) the fruit skin during this process. The remains of the bract whorl including the cut peduncle end, were sealed using a fast-curing, non-phytotoxic silicone rubber (Dowsil SE 9186 Sealant; Dow, Midland, MI, USA).

Water soaking was induced by incubating fruit individually in deionized water (one fruit per 100 mL). The fruit was forced underwater using a soft plastic-foam plug. After a pre-specified interval, a fruit was removed from the water, carefully blotted using soft tissue paper and weighed (ME235P-OCE; Sartorius, Goettingen, Germany). Water soaking was quantified using a five-point rating scale [2]. The rating scale was: score 0 = no water soaking; score 1 = <10% of the surface area water-soaked; score 2 = 10 to 35%; score 3 = 35 to 60% and score 4 = >60% of the surface area water-soaked. Earlier studies established that the water soaked area (a continuous variable) and the rating scores (a discontinuous score) were about linearly related [2].

All experiments were carried out in a temperature-controlled laboratory at 22°C. The number of individual fruit replicates per treatment was 15, unless otherwise specified.

### Experiments

#### Establishing the effect of CaCl_2_

The time course of water soaking was established in the presence and absence of 10 mM CaCl_2_. Deionized water served as control. Fruit were incubated for 0, 2, 4, 8, 16, 24, 36 or 48 h. Water uptake, water soaking and leakage of anthocyanin into the incubation solution were determined. The leakage of anthocyanin was quantified by measuring the absorbance of the incubation medium at 520 nm using a spectrophotometer (Specord 210; Analytik Jena, Jena, Germany). The pH was adjusted to 2.3 using citric acid at a final concentration of 37 mM before measuring absorbance.

The effect of the CaCl_2_ concentration on water soaking was studied by incubating fruit in solutions of 0, 1, 3, 10, 30 or 100 mM CaCl_2_. Water uptake and water soaking were quantified as described above. The amount of Ca taken up during incubation was also established. Fruit were freeze-dried for 3 to 4 d, followed by drying at 103°C for 15 d. Individual fruit dry mass was also determined. The dried samples were ground in a ball mill at 30 Hz for 10 s (MM 400 mill; Retsch, Haan, Germany). The powder was re-dried for 3 d at 103°C before an aliquot of 100 mg was taken and ashed in a muffle furnace (L24/11/B180; Nabertherm, Lilienthal, Germany) at 500°C (heating phase: from 20 to 500°C 2 h, holding phase: 4 h at 500°C). When ashing was incomplete as indexed by dark black ash, the samples were again taken up in 200 μl of 1 N HCl and re-ashed using the same settings. The ash was taken up in 2 ml of 1 N HCl plus 8 ml of deionized water and filtered (MN 640 M; Macherey-Nagel, Dueren, Germany). To eliminate interference from P in the Ca analyses, LaCl_3_ was added to the solutions at a final concentration of 1% [7]. The solution was diluted with deionized water as required to obtain a Ca concentration within the measuring window (range 0 to 4 mg l^−1^ of Ca). Samples were analyzed using an atomic absorption spectrometer (AAS) (Analyst 300; Perkin Elmer, Waltham, MA, USA) equipped with a Ca lumina hollow cathode lamp (wavelength 422.7 nm, slit 0.7 nm) using an air–acetylene flame.

Whether the effect of CaCl_2_ was specific to ‘Florentina’ strawberry was investigated by comparing different cultivars, i.e., Clery, Sonsation, Malwina, Faith. In each case fruit were incubated with or without CaCl_2_ at 10 mM for 24 h. Water uptake and water-soaking were quantified.

#### The mode of action of Ca

The effect of the chelating agent EGTA (Ethyleneglycol-*bis*(β-aminoethyl)-N,N,Nʹ,Nʹ-tetraacetic acid, CAS Nr. 67-42-5) on water soaking was established by incubating fruit in 10 mM EGTA and 10 mM CaCl_2_, or deionized water for 4 h. The chelating agent EGTA was selected because it has a high affinity for Ca and, therefore, can extract Ca from the cell wall. Water uptake and water soaking were quantified.

To establish whether a putative decrease in water soaking due to CaCl_2_ was related to the cation or the anion, two experiments were conducted. The first focused on the anions. Different organic (Ca-acetate, Ca-formate, Ca-propionate, Ca-lactate, Ca-heptagluconate) and inorganic (CaSO_4_, Ca(NO_3_)_2_, CaCl_2_) Ca-salts were compared, each at 10 mM. The second experiment focused on the cations. Here, we compared the effects of monovalent (Na^+^, K^+^, NH_4_^+^, Li^+^), divalent (Mg^2+^, Ca^2+^, Cu^2+^, Mn^2+^, Sr^2+^, Ba^2+^) and trivalent (Fe^3+^, Al^3+^) cations on water uptake and water soaking. All these cations were partnered with chloride due to their high water solubility, concentrations were all 10 mM. Deionized water served as control. The incubation period was 8 h. Water uptake and water soaking were quantified.

The effect of CaCl_2_ on microcracking was determined after incubating fruit in deionized water or in isotonic polyethylene glycol 6000 (PEG 6000) with or without 10 mM CaCl_2_ for 4 h. Isotonic PEG 6000 was used to effectively eliminate water uptake as a potential factor in microcracking. This allowed separation of the effects of surface wetness from those of water uptake. Following incubation, fruit were immersed in 0.1% of the fluorescent tracer acridine orange (Carl Roth, Karlsruhe, Germany) for 5 min, rinsed with deionized water and carefully blotted. The fruit surface was then inspected at ×3.2 under incident fluorescent light using a binocular microscope (MZ10F with filter GFP plus excitation wavelength 480–440 nm, emission wavelength ≥510 nm; Leica Microsystems GmbH, Wetzlar, Germany). Four randomly selected calibrated images were taken (Camera DP71; Olympus, Hamburg, Germany) in the region of the maximum diameter of the fruit. A total of 20 fruit per treatment were inspected. The fluorescent tracer acridine orange penetrates any microscopic cracks in the cuticle. Tissue infiltrated with acridine orange emits orange, yellow and green fluorescence [8]. The area infiltrated by acridine orange was quantified using image analysis (cellSens Dimension 2.3.1; Olympus). The infiltrated area was expressed as a percentage of the area of the microscope window (2.2 x 1.7 mm). The total number of replicates per treatment was 80.

The effects of CaCl_2_ on the integrity of the cell wall and plasma membrane were studied using anthocyanin leakage from flesh discs as an indicator [9]. A time course of anthocyanin leakage was established. Tissue cylinders were excised from the outer flesh using a biopsy punch (8 mm diameter). The skin was removed. Using parallel-mounted razor blades, cylinders were cut transversely to form 2 mm thick discs. The discs were then blotted, rinsed and incubated in isotonic PEG 6000 solution with or without 10 mM CaCl_2_ for 0, 2, 4, 8, 16 or 24 h. For sampling, discs were removed from incubation medium, the pH of the medium adjusted to pH 2.5 and the absorbance quantified at 520 nm (Specord 210; Analytik Jena, Jena, Germany). Based on these results a 24 h time interval was selected for the subsequent experiment. Here, the effects of CaCl_2_ on the bursting pressure of the cell wall were examined. Cell walls were stressed to varying extents by incubating discs in solutions of PEG 6000 at osmotic potentials of 0, -0.5, -1.0, -1.5 or -2.0 MPa with or without 10 mM CaCl_2_. Absorbance of the incubation medium was measured as described above. Six discs were excised per fruit and used as paired observations with three discs representing one replicate. The experiment was carried out using ten replicates.

### Data analyses

All experiments were conducted and analyzed using completely randomized designs. Data were analyzed by analysis of variance. Means were compared using the Dunnett test or Tukey’s studentized range tests (all p < 0.05) using R (version 4.1.0; R Foundation for Statistical Computing, Vienna, Austria), and regressions were carried out using the SAS software package (version 9.4; SAS Institute Inc., Cary, NC). Data are presented as means ± standard errors. All data shown in the Figures and Tables are available in the S1 File.

## Results

**Fig 1:**
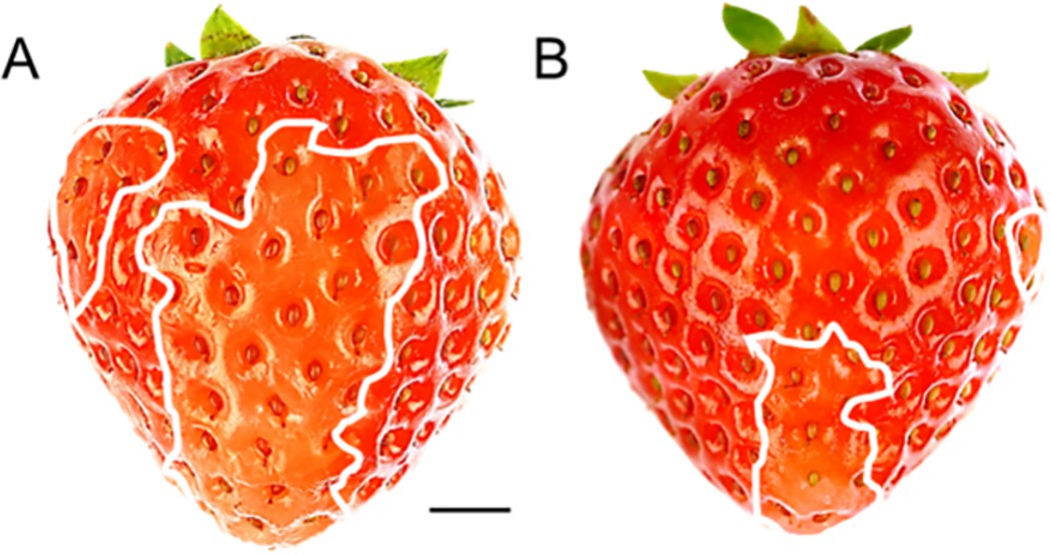
Typical symptoms of water soaking in strawberry ‘Florentina’ after incubation in (A) water (score 3) and in (B) 10 mM of CaCl_2_ (score 2) for 8 h. Water soaking was indexed using a five-point rating scale: score 0, no water soaking; score 1, < 10% of the surface area water-soaked; score 2, 10 to 35%; score 3, 35 to 60%; score 4 > 60%. The white line indicates the extent of the water-soaked area. Scale bar in A = 5 mm. The images are representative of 15 individual fruit replicates per treatment.

Water soaking appeared as irregular pale patches of deliquescent skin. These were watery, slightly translucent and dull, compared with the dark-red and shiny appearance of an adjacent intact surface on the same fruit or on a control fruit. The symptoms induced by incubation in deionized water or in 10 mM CaCl_2_ did not differ significantly, except that the affected surface areas, were markedly smaller in fruit incubated in CaCl_2_ (Fig 1).

**Fig 2:**
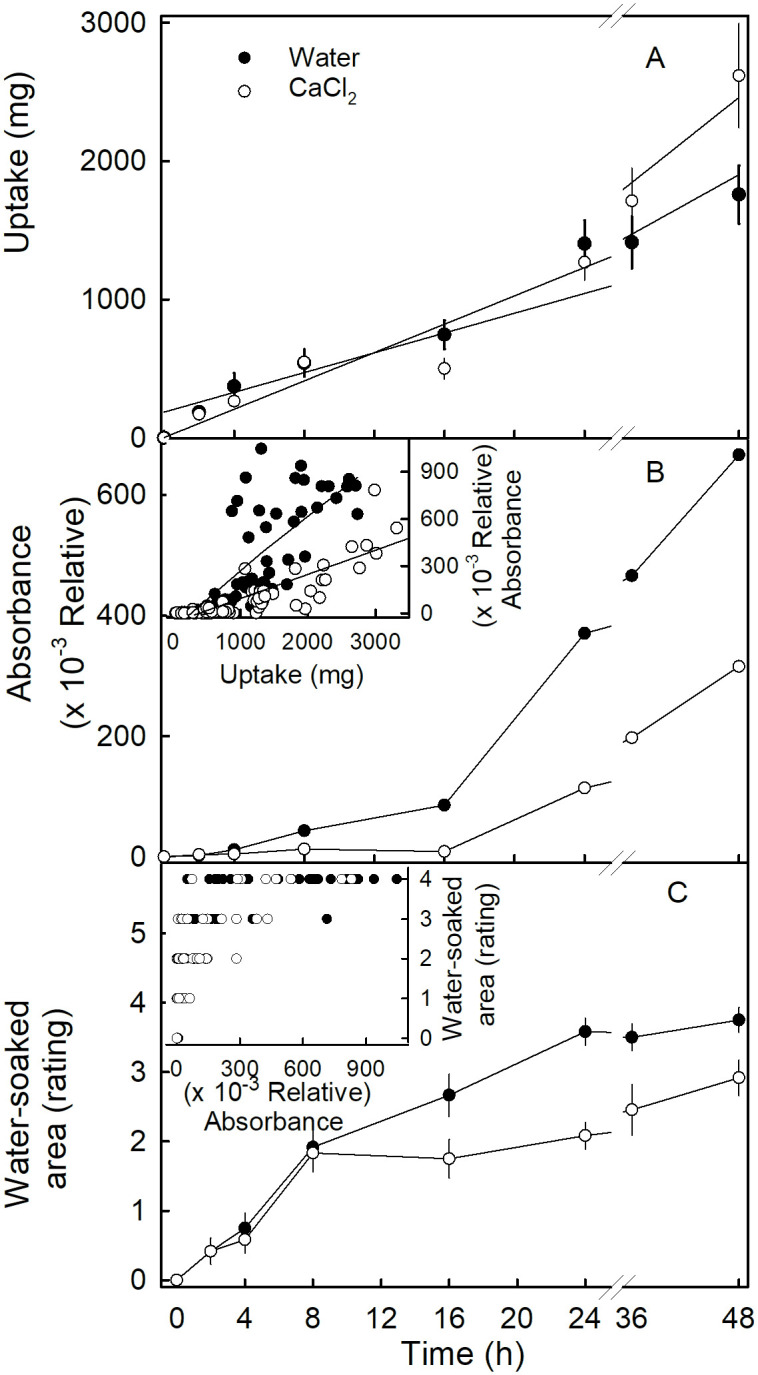
Time course of (A) water uptake, (B) leakage of anthocyanins as indexed by the absorbance (520 nm) of the incubation solution; and (C) change in area affected by water soaking. Inset (B): Relationship between anthocyanin leakage and water uptake, Inset (C): Relationship between water-soaked area and anthocyanin leakage. Strawberry fruit ‘Clery’ was incubated in deionized water or in 10 mM CaCl_2_.

Water uptake increased linearly with time. There were no significant differences in rates of water uptake, with or without CaCl_2_ (Fig 2A). However, the leakage of anthocyanin, as indexed by the absorbance of the incubation medium, increased with time in an expo-linear pattern (Fig 2B). Following an initial lag phase of about 16 h, medium absorbance increased rapidly for fruit incubated in water, but the increase was markedly less in fruit incubated in CaCl_2_. During the lag phase, leakage occurred at a low rate for fruit incubated in water, but leakage did not occur for fruit incubated in CaCl_2_ (Fig 2B). The relationship between uptake and leakage had a breakpoint that corresponded to the onset of cell bursting. At about 364 mg uptake, anthocyanin leakage began to increase for fruit incubated in water (Table 1). In contrast, in the presence of CaCl_2_, the breakpoint was increased markedly to 712 mg. Furthermore, the amount of leakage per unit of water uptake was higher for fruit incubated in water, than for fruit in CaCl_2_ (Fig 2B, inset; Table 1). Water soaking increased with time up to 8 h for fruit incubated in deionized water or in CaCl_2_. Beyond 8 h, water soaking increased at a higher rate for fruit incubated in water as compared to fruit incubated in CaCl_2_ (Fig 2C). At low levels of water soaking, the rating scores and anthocyanin leakage were positively related (Fig 2C, inset). However, as the rating scores approached a maximum, the rating became less dependent on anthocyanin leakage (Fig 2C, inset).

**Table 1:** Parameter estimates of the Goudriaan and Monteith [10] expo-linear regression model used to describe the relationship between anthocyanin leakage (absorbance) and water uptake.

Treatment	Parameter			Root mean squared error (RMSE)	P-value
	A x 10^−4^ (Relative absorbance mg^-1^)	B x 10^−2^ (mg^-1^)	K (mg)		
Control	3.8 ± 0.4	3.3 ± 32.6	364 ± 107	0.17	<0.0001
CaCl_2_	1.7 ± 0.2	0.4 ± 0.3	712 ± 168	0.08	<0.0001

The regression equation was: $Absorbance=\frac{A}{B}\times \text{ln}\left(1+e^{B\times \left(Uptake-K\right)}\right)$ where A is the slope of the linear phase representing the amount of leakage per unit water uptake, B is the maximum relative rate of the non-linear phase that represents the increase in leakage per unit of absorbance per unit water uptake, and K is the x-axis intercept the linear phase that the amount of water taken up when the bursting of cells begins.

**Fig 3:**
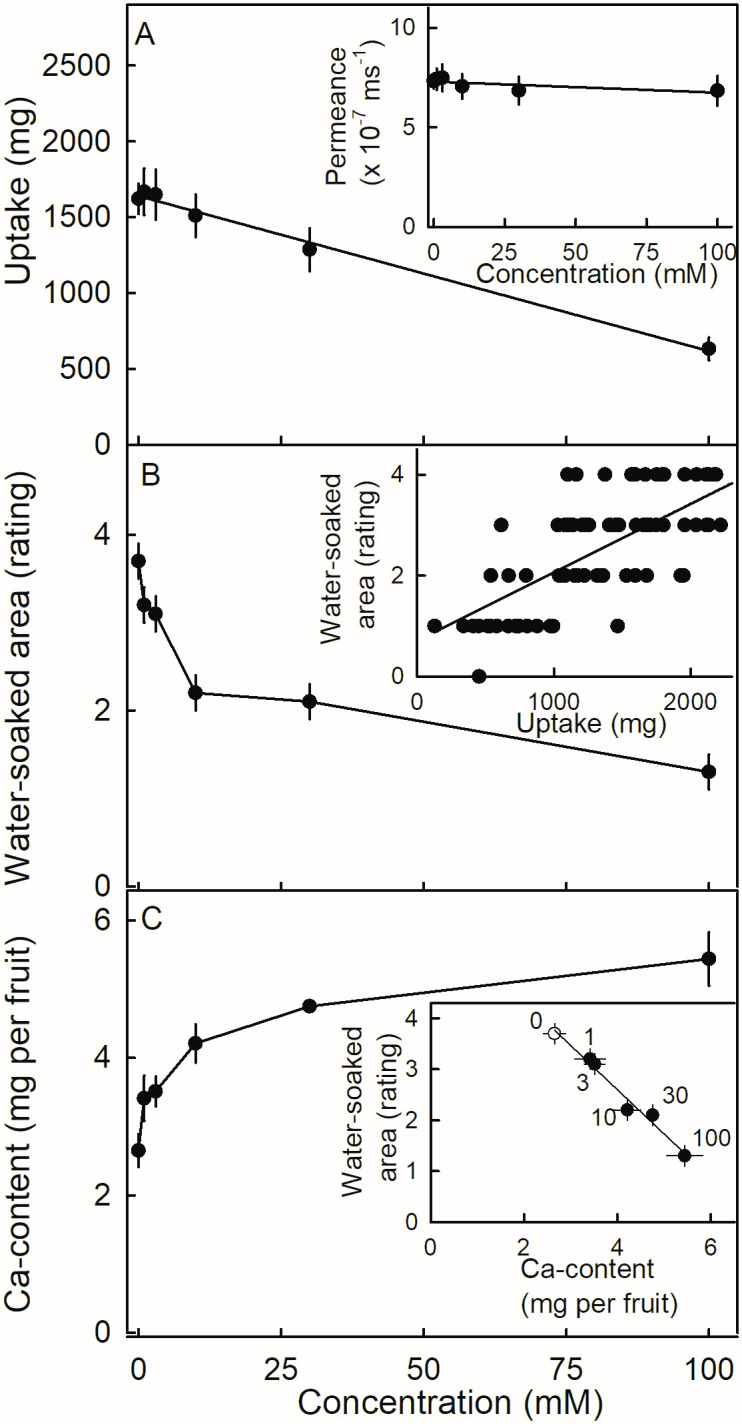
Effect of the concentration of CaCl_2_ on (A) water uptake, (B) water soaking, and (C) Ca content of ‘Clery’ strawberry. Inset in (A): Relationship between permeance and CaCl_2_ concentration. Inset in (B): Relationship between water soaking and water uptake. Inset in (C): Relationship between water soaking and fruit Ca content. The numbers on the graphs represent the corresponding CaCl_2_ concentration (mM).

Water uptake decreased linearly with increasing concentrations of CaCl_2_ (Fig 3A). There was no significant change in water permeance, indicating that the decrease in uptake was most likely an osmotic effect, and resulting from a decrease in driving force (Fig 3A, inset). Increasing CaCl_2_ concentrations decreased the water-soaked area, particularly at concentrations up to 10 mM CaCl_2_ (Fig 3B). As CaCl_2_ concentration increased, fruit calcium content increased asymptotically (Fig 3C). Water soaking and fruit calcium content were negatively and linearly related (Fig 3C, inset).

The decrease in water soaking with CaCl_2_ was not specific to just one cultivar but occurred in all cultivars investigated. In contrast, the effect of CaCl_2_ on water uptake was variable. Compared with in water, water uptake decreased in the presence of CaCl_2_ in ‘Clery’ and ‘Malwina’, whereas in ‘Sonsation’, ‘Faith’ and ‘Florentina’ water uptake increased in the presence of CaCl_2_. Interestingly, the three cultivars that had the greater water uptake in the presence of CaCl_2_ were also the ones more susceptible to water-soaking (Table 2).

**Table 2:** Effect of CaCl_2_ (10 mM) on water soaking of ripe strawberries of different cultivars. Fruit was incubated for 24 h.

Cultivar	Uptake (mg)			Water-soaked area (rating)		
	Control	CaCl_2_	Mean	Control	CaCl_2_	Mean
Clery	1069 ± 93 a^a^	721 ± 105 b	895 ± 76	3.2 ± 0.2	1.6 ± 0.2	2.4 ± 0.2 b
Sonsation	1537 ± 104 a	2463 ± 261b	2000 ± 162	3.7 ± 0.2	2.9 ± 0.3	3.3 ± 0.2 a
Malwina	1122 ± 134 a	713 ± 134 b	917 ± 100	1.7 ± 0.2	0.7 ± 0.2	1.2 ± 0.2 c
Faith	1511 ± 153 a	2339 ± 306 b	1925 ± 185	3.3 ± 0.3	2.9 ± 0.3	3.1 ± 0.2 a
Florentina	889 ± 49 a	1480 ± 129 b	1185 ± 87	3.7 ± 0.1	2.9 ± 0.2	3.3 ± 0.2 a
Mean	1226 ± 57	1543 ± 125		3.1 ± 0.1 a	2.2 ± 0.1b	

^a^ Two factorial AOV revealed significant interaction for water uptake, but not significant main effects for water soaking. Mean separation for water uptake within cultivars, mean separation for water soaking within main effects by Tukey’s studentized range test, p = 0.05. Deionized water served as control. Water soaking was indexed using a five-score rating scale: score 0, no water soaking; score 1, < 10% of the surface area water-soaked; score 2, 10 to 35%; score 3, 35 to 60%; score 4 > 60%.

Incubating fruit in EGTA markedly increased water soaking compared to in the control (water), whereas incubation in CaCl_2_ deceased water soaking (Table 3). Incubation in EGTA resulted in the highest water uptake, and that incubated in CaCl_2_ the lowest water uptake.

**Table 3:** The effect of CaCl_2_ (10 mM) and the chelating agent EGTA (10 mM) on water soaking of ‘Clery’ strawberries. The fruit was incubated for 4 h.

Treatment	pH	Uptake (mg)	Water-soaked area (rating)
Water	6.3	797 ± 89 ab	2.0 ± 0.2 b
CaCl_2_	6.7	553 ± 63 b	1.4 ± 0.2 c
EGTA	8.2	889 ± 101 a	2.9 ± 0.2 a

^a^Mean separation within columns by Tukey’s studentized range test at *p* = 0.05. Water soaking was indexed using a five-score rating scale: score 0, no water soaking; score 1, < 10% of the surface area water-soaked; score 2, 10 to 35%; score 3, 35 to 60%; score 4 > 60%.

The Ca salts differed in their effects on water soaking. Only CaCl_2_ significantly reduced water soaking, as compared to the water control. The effects of all other anions were not significant. None of the salts had a significant effect on water uptake (Table 4).

**Table 4:** Comparison of Ca salts all at 10 mM on water soaking of ‘Florentina’ strawberries incubated for 8 h.

Salt	pH	Uptake (mg)	Water-soaked area (rating)
Water	6.9	689 ± 74 ^(ns)^	2.8 ± 0.2 a^a^
CaSO_4_	6.9	445 ± 62	2.0 ± 0.2 a
Ca(NO_3_)_2_	6.8	592 ± 108	2.4 ± 0.3 a
CaCl_2_	6.7	550 ± 57	1.8 ± 0.1 b
Calcium heptagluconate	6.8	574 ± 89	2.2 ± 0.2 a
Calcium lactate	6.9	467 ± 48	2.0 ± 0.2 a
Calcium propionate	7.2	425 ± 56	2.4 ± 0.3 a
Calcium formate	6.8	485 ± 63	2.8 ± 0.2 a
Calcium acetate	7.2	520 ± 89	2.5 ± 0.2 a

^(ns)^Means do not differ significantly from the control.

^a^Mean followed by the same letter do not differ significantly from the control, Dunnett’s test at *p* = 0.05. Water soaking was indexed using a five-score rating scale: score 0, no water soaking; score 1, < 10% of the surface area water-soaked; score 2, 10 to 35%; score 3, 35 to 60%; score 4 > 60%.

**Fig 4:**
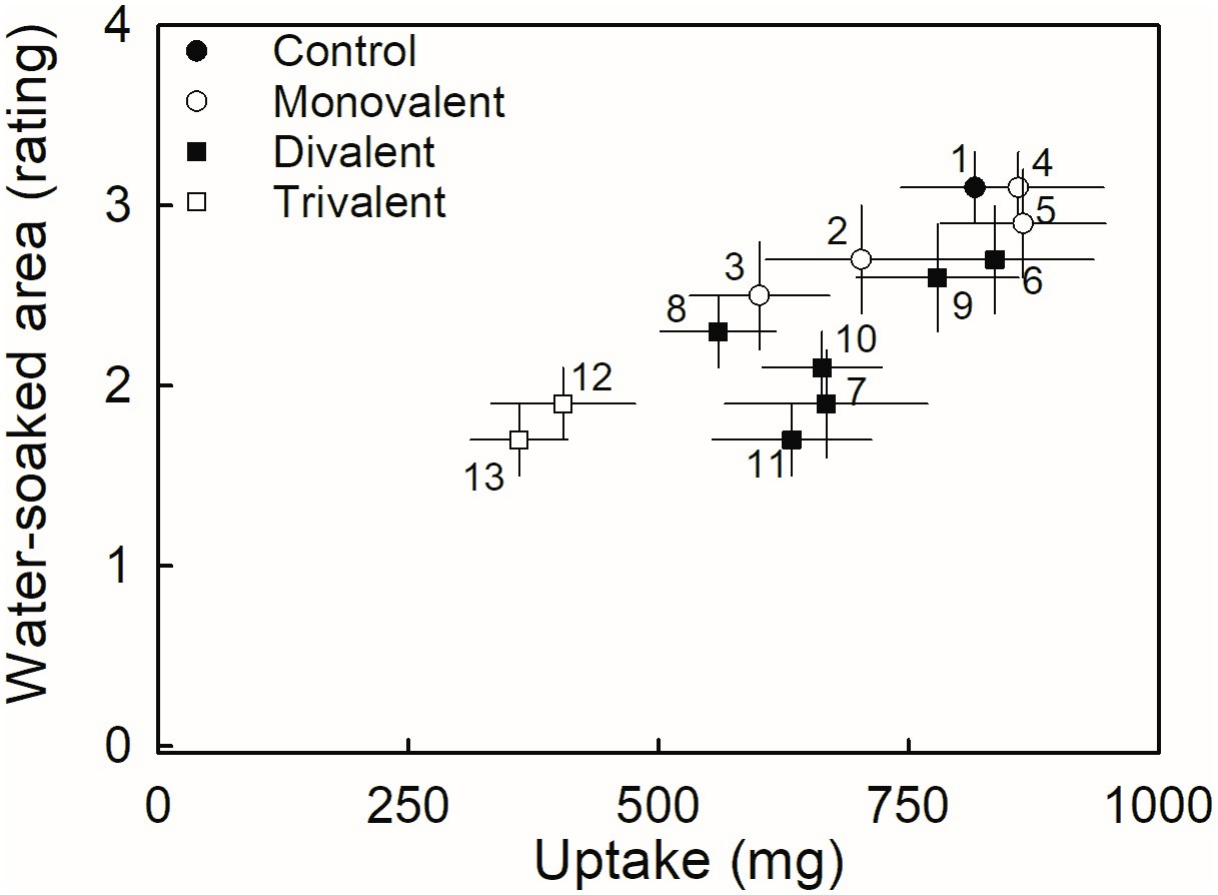
Relationship between water soaking and water uptake of ‘Florentina’ strawberry as affected by the chlorides of various cations. Fruit were incubated in chlorides of monovalent (NaCl (2), KCl (3), NH_4_Cl (4), LiCl (5)), divalent (MgCl_2_ (6), CaCl_2_ (7), CuCl_2_ (8), MnCl_2_ (9), SrCl_2_ (10), BaCl_2_ (11)) or trivalent cations (FeCl_3_ (12), AlCl_3_ (13)). Deionized water (1) served as control. The coefficient of correlation was r = 0.80***.

Comparison of the effects of the cations as chlorides, revealed that the monovalent cations had no effects, either on water soaking or on water uptake. Three out of the six divalent cations (Ca, Ba, Sr) and the trivalent cations (Fe, Al) all decreased water soaking compared to the water control. Only the trivalent cations (Fe, Al) significantly decreased water uptake compared to the control (Table 5; Fig 4).

**Table 5:** Comparison of chloride salts with different cations all at 10 mM on water soaking in ‘Florentina’ strawberry incubated for 8 h.

Salt	pH	Uptake (mg)	Water-soaked area (rating)
Water	6.9	817 ± 74 a^a^	3.1 ± 0.2 a
NaCl	7.4	703 ± 95 a	2.7 ± 0.3 a
KCl	6.8	601 ± 70 a	2.5 ± 0.3 a
NH_4_Cl	6.8	860 ± 85 a	3.1 ± 0.2 a
LiCl	7.7	865 ± 83 a	2.9 ± 0.3 a
MgCl_2_	7.2	837 ± 99 a	2.7 ± 0.3 a
CaCl_2_	6.5	668 ± 102 a	1.9 ± 0.3 b
CuCl_2_	5.1	560 ± 58 a	2.3 ± 0.2 a
MnCl_2_	6.4	779 ± 81 a	2.6 ± 0.3 a
SrCl_2_	6.4	664 ± 58 a	2.1 ± 0.2 b
BaCl_2_	5.5	633 ± 80 a	1.7 ± 0.2 b
FeCl_3_	2.4	404 ± 72 b	1.9 ± 0.2 b
AlCl_3_	4.1	360 ± 49 b	1.7 ± 0.2 b

^a^Means followed by the same letter do not differ significantly from the control, Dunnett ‘s test at *p* = 0.05. Water soaking was indexed using a five-score rating scale: score 0, no water soaking; score 1, < 10% of the surface area water-soaked; score 2, 10 to 35%; score 3, 35 to 60%; score 4 > 60%.

**Fig 5:**
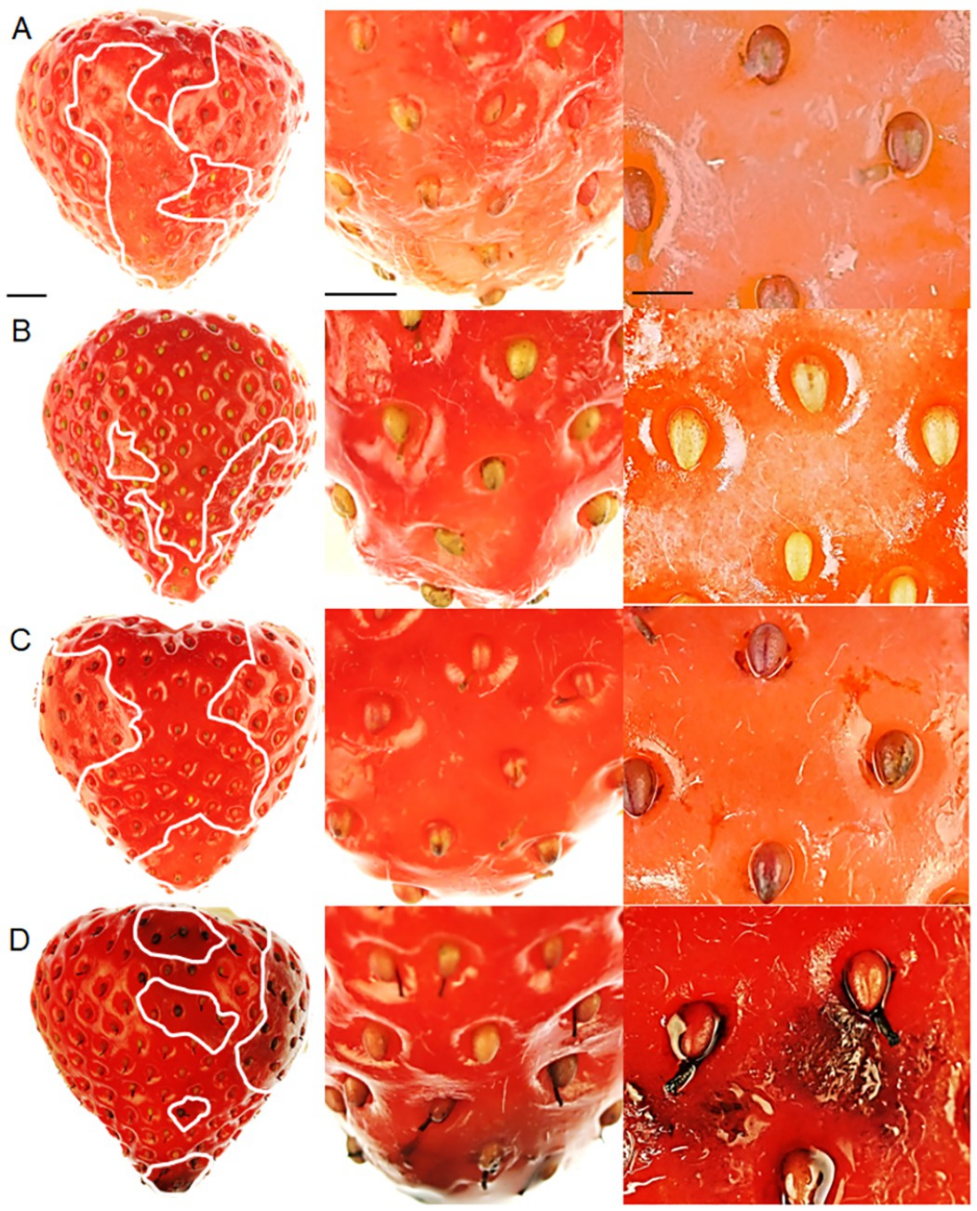
Water soaking of fruit incubated in (A) deionized water, (B) CaCl_2_, (C) CuCl_2_, and (D) FeCl_3_. The white line indicates the extent of the water-soaked area. Scale bar in A = 5 mm (top row), 2 mm (center row), 1 mm (bottom row).

It is interesting that the appearance of symptoms of water soaking changed when incubating strawberries in CuCl_2_ or FeCl_3_, but not in CaCl_2_ (Fig 5A and 5B). After incubation in CuCl_2_, the water soaked tissue was opaque and reddish (Fig 5C). Fruit incubated in FeCl_3_ had numerous localized black-colored precipitates particularly in the depressions around the achenes (Fig 5D).

Aqueous CaCl_2_ also decreased microcracking of the strawberry cuticle as compared to incubation without CaCl_2_. However, when fruit was incubated in isotonic PEG 6000 in the presence of CaCl_2_, water uptake was markedly reduced, and there was no effect of CaCl_2_ on water soaking or on microcracking (Table 6).

**Table 6:** Effect of CaCl_2_ (10 mM) on water uptake, water soaking and microcracking of the cuticle of ‘Florentina’ strawberry.

Incubation	Uptake (mg)	Water-soaked area (rating)	Microcracking area (%)
None	-	0.0 ± 0.0 c	6.7 ± 0.8 a
Water	554 ± 79 a^a^	2.0 ± 0.2 a	31.7 ± 3.7 c
Water+ CaCl_2_	434 ± 41a	1.4 ± 0.2 b	17.3 ± 2.8 b
Isotonic PEG 6000	165 ± 23 b	0.2 ± 0.1 c	12.3 ± 1.6 b
Isotonic PEG 6000+ CaCl_2_	135 ± 18 b	0.2 ± 0.1 c	12.5 ± 1.2 b

^a^Mean separation within columns by Tukey’s studentized range test at *p* = 0.05. Microcracking area (%) was indexed as the area infiltrated by aqueous acridine orange. Acridine orange penetrates the strawberry fruit skin via microcracks in the cuticle. Non-incubated fruit served as control. Water soaking was indexed using a five-point rating scale: score 0, no water soaking; score 1, < 10% of the surface area water-soaked; score 2, 10 to 35%; score 3, 35 to 60%; score 4 > 60%.

**Fig 6:**
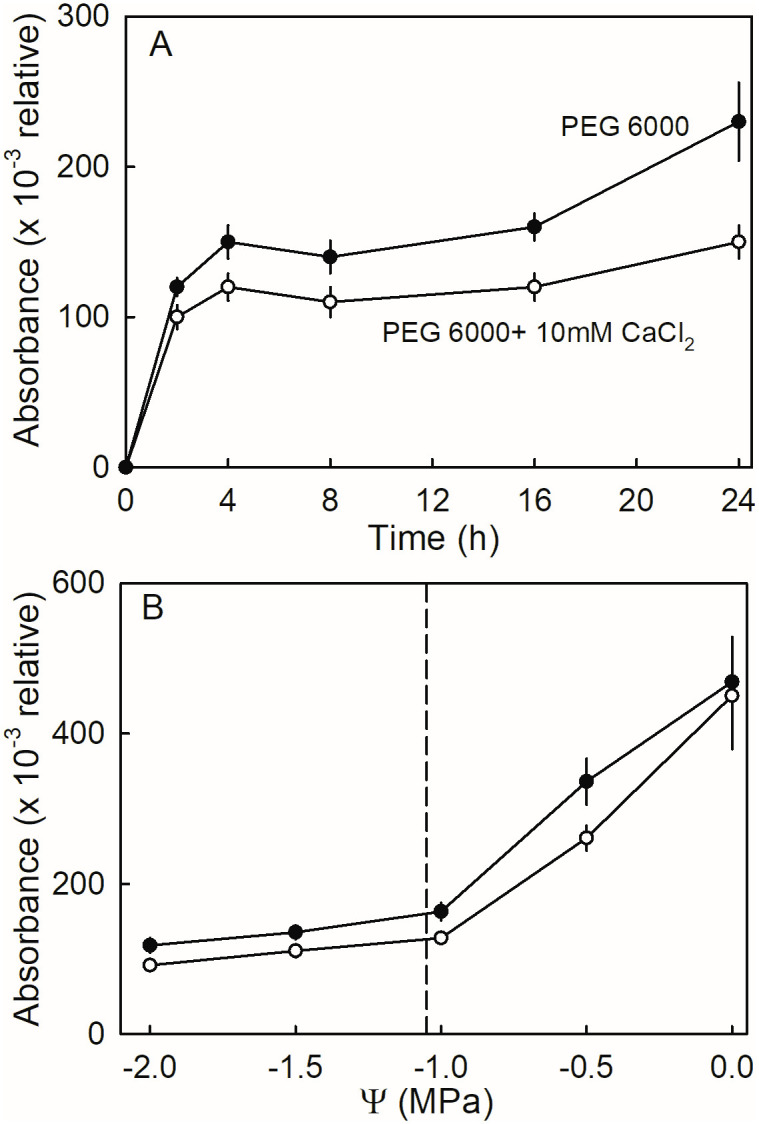
(A) Time course of anthocyanin leakage from flesh discs excised from ripe ‘Clery’ strawberries in the presence or absence of CaCl_2_. Flesh discs were incubated in isotonic polyethylene glycol 6000 (PEG 6000) solutions. (B) Effect of osmotic potential (Ψ) of the incubation solution on anthocyanin leakage in the presence or absence of CaCl_2_. The vertical dashed line indicates the osmotic potential of juice expressed from fruit of the same batch.

Incubating flesh discs in isotonic PEG 6000 solution, with or without 10 mM CaCl_2_, rapidly increased leakage of anthocyanin up to about 4 h, thereafter leakage was at a lower rate (Fig 6A). Discs incubated in CaCl_2_ had lower anthocyanin leakage than the control. When varying the osmotic potential of the PEG 6000 solution in the presence or absence of CaCl_2_, anthocyanin leakage increased as osmotic concentration increased, particularly beyond isotonicity, when the osmotic concentration of the incubation solutions exceeded that of the fruit’s juice (i.e., the solution osmotic potential became more negative) (Fig 6B). Irrespective of osmotic potential, CaCl_2_ reduced the leakage of anthocyanins. There were no significant interactions between the two factors.

## Discussion

In our discussion we will focus on (1) the decrease in water soaking due to the presence of Ca salts and (2) the effects of the Ca salts on water uptake.

### Calcium decreases water soaking

Calcium, and some of the other divalent and trivalent cations decreased water soaking. Accordingly, extraction of Ca by incubation in the chelating agent EGTA increased water soaking. Due to the complex nature of water soaking according to the Zipper model, one or several of the component processes leading to water soaking must have been affected by Ca.

First, Ca decreased microcracking of the cuticle. This effect was significant only when the Ca solution was not osmotically buffered using PEG. This observation indicates that Ca most likely increased the crosslinking of the underlying cell walls that are probably causal in cracking of the cuticle. This is consistent with (1) the extremely thin and fragile cuticle of the strawberry fruit [4] and (2) numerous studies reporting increased crosslinking of cell walls by Ca and other divalent or trivalent cations [6, 11]. Pectins are major constituents of cell walls and carry a negative charge at physiological values of pH. Cations bind to these negative charges and di- and trivalent cations thereby increase crosslinking. Calcium also decreases pectin solubilization [6, 12]. Similar effects on crosslinking have been reported for FeCl_3_ in cracking of sweet cherry fruit [13]. The effect of Ca on crosslinking of cell walls in strawberry was statistically significant, but not very large. In the presence of Ca, the amount of water uptake tolerated without bursting of cells and leakage of anthocyanins was higher compared to the amount taken up in the water control. Moreover, the amount of leakage per unit uptake was markedly reduced in the presence of CaCl_2_. Meanwhile, when incubating flesh discs in hypertonic and hypotonic solutions with and without Ca, the Ca effect was not affected by the tonicity of the incubation solution. If Ca had a pronounced effect on crosslinking of cell walls, we would expect the Ca effect to be larger in hypotonic than in hypertonic solutions. When incubated in hypotonic solutions, we would expect water uptake to strain the cell walls, and eventually lead to cell bursting. This would not be the case during incubation in hypertonic solutions.

Second, Ca reduced the amount of leakage of anthocyanin, indicating that the semipermeability of plasma membrane and tonoplast were better maintained in the presence of Ca, than in its absence [14]. Similar effects have been reported for the membrane permeabilities in apples and tomatoes [15–17]. Since anthocyanin leakage was reduced in the presence of CaCl_2_, we also expect less leakage of citric and malic acids. The leakage of these organic acids triggers the chain reaction that causes neighboring cells to collapse and thus also to release their organic acids [18]. In strawberries, this reaction results in a tangential spreading of the water-soaked area over the fruit surface and also radially, deeper into the flesh [2]. Less leakage in presence of Ca therefore implies less water soaking.

### Effects of Ca and other cations on water uptake

Calcium had no consistent or specific effects on water uptake. The effect of Ca was purely osmotic and, hence, the result of a decrease in driving force for osmotic water uptake. Assuming an osmotic potential of strawberry juice of -1.06 MPa, a 10 mM CaCl_2_ solution (osmotic potential -0.04 MPa) would account for about a 4% reduction in water uptake rate due to a decreased osmotic driving force. This increases to a 58% reduction in driving force for 100 mM CaCl_2_ (osmotic potential -0.62 MPa). Correcting for the change in driving force due to CaCl_2_ reveals the permeance of the cuticle to water remained constant and independent of CaCl_2_ concentration. This is consistent with earlier findings for water uptake into sweet cherry fruit [13]. This explanation also applies to the chlorides of other divalent cations. For monovalent cations, the reduction in driving force will be even smaller due to their less negative osmotic potentials [13]. It is important to note that Ca may indirectly affect the change in fruit mass associated with water uptake due to effects on microcracking and on leakage of cell contents, and particularly so after the longer incubation periods.

The effects of the trivalent chloride salts, FeCl_3_ and AlCl_3_, on water uptake must have a different basis. Both markedly reduced water uptake at 10 mM. Although their osmotic potentials were more negative than those of the divalent chlorides, the decrease in water uptake cannot be accounted for by the decrease in driving force. Both salts must instead have had their effects via a change (a decrease) in the permeance of the fruit skin. This conclusion is consistent with findings reported for sweet cherry [19, 20]. These authors attributed the decrease in permeance of sweet cherry fruit skin to a plugging of the ‘rapid-penetration’ pathways that bypass the cuticle barrier [19, 20]. This plugging is due to a precipitation reaction as a result of the increase in pH encountered during penetration [20]. Aqueous solutions of both salts are highly acidic (their pHs are very low, in the range of pH 1.7 to pH 3.6 [19]. When encountering the higher pH of the apoplast, viscous oxides and hydroxides are formed that precipitate and plug the rapid-penetration pathways across the cuticle. As a consequence, the permeance of the fruit skin to water decreases. Support for this interpretation comes also from the black (ferrous) precipitates that formed when strawberries were incubated in the FeCl_3_ solutions. Thus, water soaking decreased due to the decreased water uptake. Unfortunately, the effects of FeCl_3_, or of other ferric salts, or of AlCl_3_ are of no practical value in horticulture due to their unacceptable ecotoxicological profiles.

## Conclusion

Our results indicate that Ca and, among the Ca salts investigated, CaCl_2_ was most effective in reducing water soaking. The mechanism through which Ca reduces water soaking is via a decrease in cuticular microcracking, hence, a decrease in leakage through the plasma membrane and tonoplast and an increase in the crosslinking of cell wall constituents.

Due to the high economic importance of water soaking in field grown strawberry production, the effects reported here warrant further study. In particular, it would seem worthwhile to explore cultural ways through which strawberry Ca content could be increased. There being no Ca translocation via the phloem [21], Ca must be imported into developing strawberries only via the xylem. Furthermore, recent investigations show that, like many other fruit species, strawberries suffer a decrease in xylem conductance during development [22]. This renders increased Ca fertilization via the soil unlikely to be effective in increasing the supply to the fruit. Consequently, Ca spray applications directly to the developing fruit remains as the only alternative. To our knowledge, this avenue for mitigating water soaking in strawberry has not been studied in greater detail. Also, uptake of Ca salts into strawberry fruit following spray application has only been addressed in a limited number of studies [23–25]. Potential benefits of an increase in fruit Ca will not be limited to a decrease in water soaking, but will also improve shelf life and fruit quality [26, 27].

## Supporting information

S1 File This is the excel file containing the data in Figs 2–4, 6 and Tables 1–6. (XLSX)
